# Novel Na_V_1.6‐Mitochondrial Complexes in Retinal Astrocyte Pockets Sequester GABA

**DOI:** 10.1002/glia.70238

**Published:** 2026-09-23

**Authors:** Joseph Matthew Holden, David John Calkins

**Affiliations:** ^1^ Department of Ophthalmology and Visual Sciences Vanderbilt University Medical Center Nashville Tennessee USA

## Abstract

Voltage‐gated sodium channels are best known for their capacity to generate and propagate electrical currents in neurons and muscle. Their expression in glial cells may also be of great importance given these cells' involvement in various disease processes. Here we identify a previously unrecognized voltage‐gated sodium channel Na_V_1.6‐enriched microdomain in mouse retinal astrocytes. Using super‐resolution confocal microscopy, electrophysiology, and transcriptomic analyses, we show that Na_V_1.6 forms high‐density clusters within intracellular compartments devoid of the calcium‐binding protein, S100β, typically understood to distribute throughout the entire astrocyte. These Na_V_1.6 pockets are polarized, clustering only on the bilayer facing retinal ganglion cell neurons, and are packed with unusually large, anaplerotic, and relatively depolarized mitochondria that closely appose Na_V_1.6 puncta. These metabolic compartments also accumulate high levels of GABA. Together, our findings reveal a specialized Na_V_1.6‐mitochondrial complex in retinal astrocytes that expands the functional repertoire of voltage‐gated sodium channels beyond direct neuronal electrical excitability.

## Graphical abstract

- Na_V_1.6 localizes in retinal astrocytes' high density pockets on the bilayer facing the ganglion cell layer.
- Na_V_ colocalizes with large, anaplerotic mitochondria.
- These microdomains accumulate high levels of GABA.

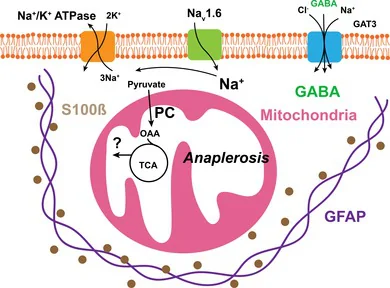

## 1 Introduction

Voltage‐gated sodium channels (Na_V_) form a family of transmembrane proteins defined by their voltage‐sensitivity and selective permeability to sodium. While their role in supporting electrogenesis in neuronal (Na_V_1.1–1.3 and Na_V_1.6–1.9) and muscle (Na_V_1.4–1.5) tissue is well‐established, non‐canonical roles for these channels have emerged in cells traditionally viewed as non‐excitable, including astrocytes (Black and Waxman 2013). Voltage‐dependent sodium currents have been characterized in astrocytes across several species and brain regions, but much of this work relies on in vitro cultures (Sontheimer et al. 1994; Glassmeier et al. 1994; Rose et al. 1997; Pappalardo et al. 2014). Consequently, an important question is to what degree the properties observed in vitro represent what occurs in vivo. While it is possible that the act of culturing astrocytes induces the expression of Na_V_s, electrical recordings in tissue slices have shown similar voltage‐sensitive sodium conductance, which increases confidence that these currents are relevant physiologically in vivo (Chvátal et al. 1995). Moreover, immunohistochemical detection of Na_V_s in astrocytes appears highly dependent on brain region, developmental state, and presence of injury or disease (Ahmad et al. 2025; Black et al. 2010; Li et al. 2025; Thompson et al. 2022).

In our recent paper, we observed that astrocytes in the mouse retina can depolarize to both onset and offset of middle‐wavelength (525 nm) light (Holden, Boal, et al. 2025). We initially investigated whether this response was due to uptake of potassium released during action potentials from retinal ganglion cells (RGCs). We treated ex vivo retina with anhydrotetrodotoxin (aTTX) to block voltage‐gated sodium channels (most robustly, Na_V_1.6 and, to a lesser extent, Na_V_1.1) and eliminate RGC spiking. Surprisingly, the astrocyte light response we described was resistant to aTTX, remaining relatively intact even after a period that was four‐fold the exposure time required to abolish RGC spiking. However, in that data, we noted (1) the average astrocyte light response amplitude decayed slightly with aTTX exposure, and (2) the resting membrane potential depolarized. These data raise the question of whether such responses are due to perturbations in the surrounding circuitry or if mouse retinal astrocytes themselves express voltage‐gated sodium channels that would be sensitive to aTTX.

Here, we conducted super resolution confocal microscopy to investigate expression of the voltage‐gated sodium channel Na_V_1.6 in retinal astrocytes and found that it localizes at high density in a node‐like distribution within astrocyte domains void of S100β, a small calcium‐binding protein generally understood to distribute throughout the entire astrocyte. The distribution is polarized in that channels are concentrated on the plasma membrane facing the RGCs and their axons but absent on the membrane facing the vitreous body of the eye. Notably, we report these domains are filled with large, anaplerotic mitochondria which sequester GABA and are in close apposition to the Na_V_ channels.

## 2 Methods

### 2.1 Animals

Strains of mice purchased from Jackson Labs include: MORF3 (035403), GFAP Cre 77.6 (024098), VGLUT2 IRES Cre (028863), and floxed tdTomato (007914). Mice used in this paper include progeny from the cross of MORF3 with GFAP Cre (G‐MORF), floxed tdTomato with GFAP Cre, and MORF3 with VGLUT2 IRES Cre. All animals used in this study were adult mice (2–7 months) with equal numbers of males and females. Animals were housed at the Vanderbilt University Division of Animal Care facility and subjected to a 12‐h light/dark cycle. Animals were provided with water and rodent chow ad libitum.

### 2.2 Immunohistochemistry and Imaging

Mice were deeply anesthetized with pentobarbital and euthanized by transcardial perfusion of phosphate‐buffered saline (PBS). Eyes were enucleated and the retina dissected as unfixed tissue. A solution of collagenase (LS005273, Worthington Biochemical) and hyaluronidase (LS002592, Worthington Biochemical) was incubated at room temperature for 10 min to aid in vitreous removal. Tissue was subsequently fixed in a solution of 4% paraformaldehyde for 1 h before washing in PBS and stored at 4°C until use. Retinas were stained as previously described in Holden et al. (2023). Briefly, blocking occurred for 3 h at room temperature in a solution of 5% normal donkey serum (NDS) and 0.1% Triton‐X. Primary antibody incubation occurred for 3 days at 4°C with gentle agitation in solutions of 0.1% Triton‐X, 3% NDS, and 1:500 dilutions of appropriate antibodies. Secondary antibody incubation occurred at room temperature with gentle agitation for 3 h in a solution of 0.1% Triton‐X, 1% NDS, and 1:200 dilutions of appropriate fluorescent antibodies. Primary antibodies used and their sources include: Bethyl [V5: A190‐120A], Abcam [V5: ab95038, S100β: ab52642, NCX‐1: ab2869], Millipore Sigma [Beta (iii) Tubulin: MAB5564, S100β: S2532, Na_V_1.6, AB5580, GABA: A2052], Invitrogen [Lucifer Yellow: A5750], Biolegend [SMI‐31: 801601], Alomone Labs [Na_V_1.6: ASC‐009, Na_V_1.1: ASC‐001‐GP, Na_V_1.8: ASC‐016], Novus [Na_V_1.6: H00006334‐M04, EAAT2: NBP1‐20136SS], Proteintech [Pyruvate Carboxylase: 16588‐1‐AP, NCBe1: 11885‐1‐AP, ATP1a1: 83191‐6‐RR, NCLX: 21430‐1‐AP], Cell Signaling [GSK3β: 9315S, EAAT1: D44E2, Calmodulin: 4830S]. We also used streptavidin conjugated to Alexa 647 from Invitrogen (S21374). Secondary antibodies were from Jackson ImmunoResearch with donkey host against IgG (H + L) for respective targets.

### 2.3 Electrophysiology

Electrical recordings and intracellular dye filling were performed as previously described (Holden, Boal, et al. 2025; Holden et al. 2023). Importantly, liquid junction potential was not corrected for in order to stay consistent with other papers from our lab. Numbers in this paper can be corrected by subtracting 13.6 mV (calculated using LJP tool in Clampfit 10.7). GABA recordings were obtained by washing on 1 mM GABA in Ames' media. Action potential shape was analyzed during periods where astrocytes were depolarized and compared to periods of no depolarization. Spikes were automatically detected in a Python script looking for a peak prominence of > 20 mV. With the spike centered at time = 0, a 2 ms window on either side of the spike was extracted. This data was shifted to a common starting voltage of 0 mV and averaged across all spikes in each respective category and plotted together.

### 2.4 Mitochondrial Potential

Relative mitochondrial membrane potential was determined using a combination of mitochondrial dyes: potential‐sensitive MitoTracker Deep Red FM and the potential insensitive Mito‐ID Green. Mito‐ID Green was used as a measure of overall mitochondrial biomass to use as a normalizing factor. Relative potential was assessed as the ratio of MitoTracker Deep Red FM to Mito‐ID Green. The retina from GFAP‐Cre/tdTomato positive animals was dissected as described for electrophysiology experiments. Dissection occurred in a dark room using red headlamps. Ames' media was used throughout the dissection process. Retinas were incubated with collagenase/hyaluronidase in a chamber with carbogen flow for 10 min before vitreous removal. After removal of vitreous, either a 1× solution of Mito‐ID Green in Ames' media or 500 nM MitoTracker Deep Red FM in Ames' media was applied to the retina for 30 min under constant flow of carbogen. Retinas were washed 3× in carbogen‐saturated Ames' and wet‐mounted on a tissue slide with spacer. A coverslip was added and additional media was introduced by capillary action. The coverslip was quickly super‐glued in place and the retina imaged within 10 min on a Nikon SoRa confocal microscope at 280×. Fluorescence was quantified in both threshold‐masked pockets and pockets demarcated by hand‐drawn borders in non‐thresholded images. We used ImageJ manual threshold of the inside of the border‐traced outline, with threshold taken to maintain features of the full‐color image.

### 2.5 Na_V_ Quantification

Retinae were immunolabeled for Na_v_1.6 using three antibodies against two distinct epitopes. The Novus antibody targets the C‐terminal region and the Millipore/Alomone Labs antibodies target the cytosolic loop between domains II and III. Confocal images at 280× with SoRa super resolution were acquired. Single plane images were taken and puncta for each antibody were counted within tdTomato/S100β—negative regions with biotin. For comparisons to neurons, a biotin signal deeper in the retina with no nearby tdTomato/S100β was used, with a circular ROI equal to the average size of the astrocyte pockets quantified.

### 2.6 3D Protein Visualization

Crystal, cryo‐EM, and AlphaFold structures of Na_v_1.6 (pdb_00008gz1, AF‐Q9WTU3‐F1), calmodulin IQ domain (pdb_00003wfn), and GSK3β (AF_AFQ9WV60F1) were obtained from RCSB Protein Data Bank. .CIF files were imported into PyMOL for visualization. Docking of Na_v_1.6 to GSK3β was predicted using Haddock 2.4 using residues 1898 and 288 in Na_V_1.6 and GSK3β, respectively, as active interacting residues. These residues were chosen based on the work of Baumgartner et al. (2025).

### 2.7 Density Analysis

Puncta for a variety of markers were analyzed for their spatial density. This was accomplished using a custom ImageJ macro and Python script. The ImageJ macro used the “Find Maxima” function to find coordinate locations of each point. A grid of coordinates was initialized in a Python script, and each point was weighted by fluorescence intensity. The density image was computed as a Gaussian blur with a sigma of 20 for this weighted grid. The blur matrix was normalized to a range of 0–1 and mapped to a jet colormap.

### 2.8 scRNA Seq and Transporter Analysis

scRNA sequencing data from the Mouse Retinal Cell Atlas (Li et al. 2024) was downloaded through the NCBI Gene Expression Omnibus (GEO, GSE243413) and Broad Institute Single Cell Portal as a pre‐processed countmatrix.h5ad file (Li et al. 2024). The h5ad file was examined using the R package Seurat and the Python package Scanpy. Cell types were already clustered by the authors, but we verified that astrocytes were GFAP and S100β‐positive cells. Gene expression data for astrocytes was filtered and reported as dot plots. Along with individual genes of interest, all transporters and channels permeable to sodium were found using HUGO Gene Nomenclature Committee (HGNC) tables of solute carrier genes coupled with Uniprot databases to determine ionic permeability. These data were manually examined to identify all channels permeable to sodium.

### 2.9 Bulk RNA Analysis

Bulk RNA sequencing files from Cullen et al. (2024) for both whole retina and micro‐dissected GFAP‐Cre Ribotag astrocytes were downloaded from NCBI's Sequence Read Archive (SRA) using the SRA toolkit (Cullen et al. 2024). SRR28818863–SRR28818874 were downloaded and converted to FASTQ. GRCm39 reference genome, transcriptome, and annotation features were downloaded from NCBI. Salmon version 1.10.2 was used on these files and the FASTQ files from Cullen et al. to quantify transcripts in a quasi‐alignment approach. The salmon output files quant.sf were parsed in Python for mouse transcripts of SCN8A (Na_V_1.6) found using the NCBI database. Transcripts per million (TPM) were recorded for each transcript and plotted in GraphPad Prism.

### 2.10 Node Detection and Quantification

A total of 87 *Z*‐stack images (249 × 249 μm^2^) of Na_V_1.6 channel (Antibody against Na_V_1.6 targets residues 1042–1061 between domains II and III) were taken at 60× magnification on a SoRa spinning disk confocal microscope at various retinal eccentricities. Images were collapsed using the standard deviation stacking method in Fiji ImageJ. Nodes were automatically detected as particles with a size between 1.25–10.00 μm^2^ and circularity between 0.35–1.00. Eccentricity was determined by linear distance to the optic nerve head. Linear regression was performed in GraphPad Prism 10.

### 2.11 Figures and Statistics

Figures were generated using Adobe Illustrator and statistics with GraphPad Prism 10; specific tests and their results are described in figure legends where relevant.

## 3 Results

**FIGURE 1:**
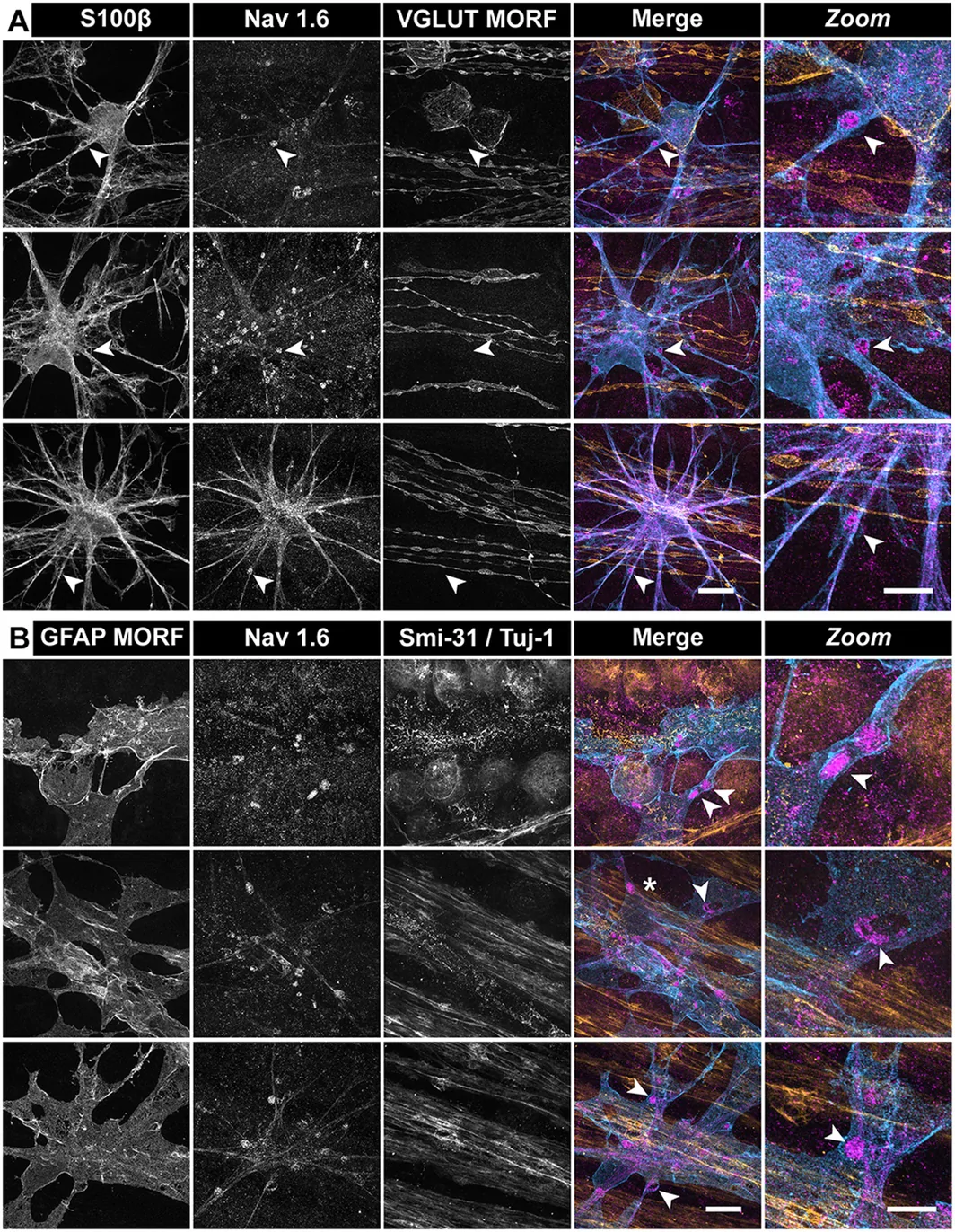
Na_V_1.6 localizes to astrocyte pockets devoid of S100β, not nearby axons. (A) S100β labels all retinal astrocytes. VGLUT MORF transgenic protein product stochastically labels complete morphology of neurons in the ganglion cell layer. Each row shows a region of the Nerve Fiber and Ganglion Cell Layers at 280× magnification with astrocytes labeled and a subset of ganglion cell bodies and axons. S100β‐negative regions of astrocyte are pockets of Na_V_1.6 labeling (arrows indicate example regions). Notice Na_V_1.6 does not localize to varicosities in RGC axons revealed by VGLUT MORF labeling. (B) Labeling of Nav1.6 colocalizes with GFAP‐MORF labeling and is spatially distinct from the pan‐axonal labeling of combined Smi‐31 and Tuj‐1 (arrows). Additionally, in some regions, the orientation of elliptical Na_V_1.6 labeling does not follow the angle of the axon bundles which would be expected if it were in an axonal node (* Row 2). Each row shows a 280× image of a stochastically‐labeled astrocyte (GFAP MORF), co‐labeled with Na_V_ 1.6 and Smi‐31 and Tuj‐1. Scale is 10 μm for full‐sized images and 5 μm for zoomed images.

In our previous paper, we reported that astrocytes depolarize to light, which decays over a lengthy time in the presence of aTTX (Holden, Boal, et al. 2025), which predominantly antagonizes Na_V_1.6. We find that retinal astrocytes do in fact express Na_V_1.6 across the entire cell, with a notable caveat. The most striking feature of this labeling is that Na_V_1.6 puncta coalesce at high density in regional nodes or pockets that are completely lacking S100β localization (Figure 1A). These Na_V_1.6‐enriched structures are roughly the shape of varicosities in RGC axons, which are known to contain a variety of channels and transporters to support action potentials. To ensure this observation was not simply attributable to axonal Na_V_1.6, we used a transgenic mouse line to stochastically label full axonal morphology. This was accomplished by crossing VGLUT‐Cre and MORF3 mice together. In Figure 1A, arrows indicate exemplary Na_V_1.6 labeling that does not overlap with labeled axons or their varicosities. However, because the MORF3 line is stochastic in its labeling, we additionally labeled for the pan‐axonal markers SMI‐31 and β(III)‐Tubulin (Tuj‐1, Figure 1B). We performed this labeling in our previously described G‐MORF line, which is analogous to the VGLUT‐Cre/MORF3 cross except the Cre recombinase is driven by a GFAP promoter (Holden et al. 2023; Holden, Bossardet, et al. 2025). In these retinas, we observe that Na_V_1.6 signal is found in membrane‐bound regions of astrocytes and does not colocalize necessarily with axonal features. Additionally, in astrocytes that overlay axon bundles, the major axis of elliptical‐shaped Na_V_1.6 signal is parallel with astrocyte processes, not axons Figure 1B (Row 2, *).

**FIGURE 2:**
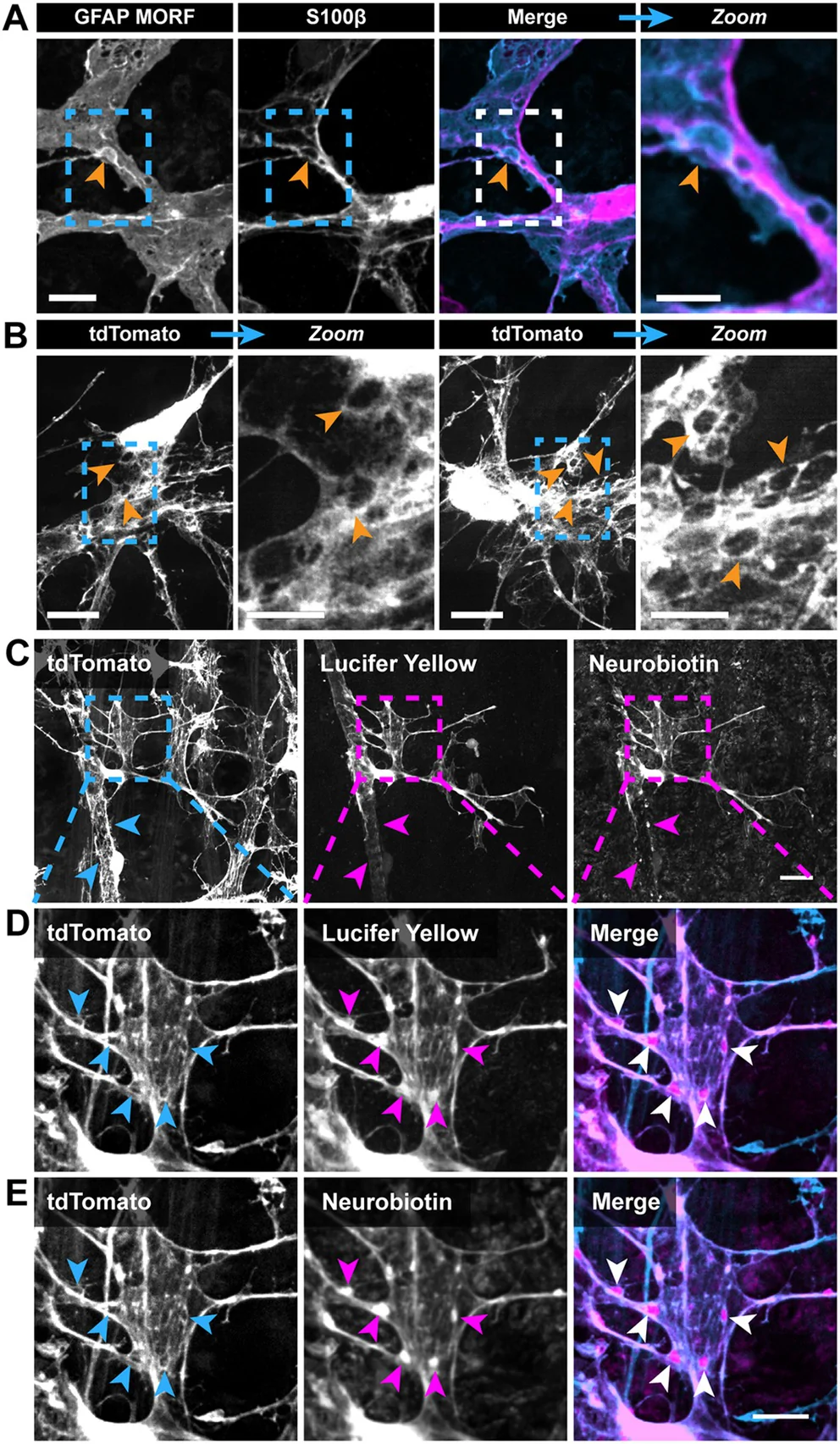
Astrocyte pockets are accessible and bound cytoplasmic compartments. (A) Full membranous morphology of a stochastically‐labeled astrocyte (GFAP MORF) co‐labeled for the cytosolic protein S100β and imaged at 60×. Arrows indicate a region of astrocyte beaded membrane that lacks S100β. (B) GFAP‐driven expression of cytosolic tdTomato reveals a similar void‐labeling pattern as S100β. Scale is 10 μm for full‐size images and 5 μm for zoomed regions in A–B. (C–E) Cells patched with small molecule dyes like (D) Lucifer Yellow and (E) Neurobiotin fill the tdTomato‐void pockets (D/E, arrows). Both Lucifer Yellow and Neurobiotin are gap‐junction permeable, but no coupled cells are observed in this example. Neurobiotin/streptavidin reaction reveals additional pocket‐like labeling not shown by Lucifer Yellow (C, arrows). This suggests the streptavidin reaction reveals endogenous biotin, perhaps in pockets of adjacent astrocytes. Scale is 20 μm for full‐size images in A–B and 10 μm for zoom and C–E.

We previously described how S100β labeling does not reveal the complete morphology of retinal astrocytes compared to membrane labeling revealed using the G‐MORF construct (Holden et al. 2023), including the presence of apparent “holes” devoid of S100β. Most of these regions are not true holes in the astrocyte membrane; they correspond to the membranous pockets that label intensely for Na_V_1.6 (Figure 2A,B). Transgenic cytosol‐directed fluorescent proteins like tdTomato reveal the same cavernous labeling of astrocyte structure as S100β, which raises the question of whether these Na_V_1.6‐enriched regions are restrictive of what can diffuse in or out of them. Since S100β and tdTomato are both cytosolic proteins, it is possible that they are too large. To test whether Na_V_1.6‐enriched pockets represent freely accessible compartments, we achieved whole‐cell configuration patch‐clamp seals of astrocytes in wholemount retinal preparations maintained ex vivo with a pipette intracellular solution containing the small molecule tracers Lucifer Yellow (LY) and Neurobiotin (NB). We find that tdTomato‐negative pockets indeed fill with both LY and NB (Figure 2C–E), implying the compartments are accessible to the rest of the cytoplasm but may restrict large protein entry. Both LY and NB are gap‐junction permeable; however, the example cell patched does not show coupled cells through the LY channel (Figure 2C–E). However, additional pocket‐like labeling is observed in adjacent cells through the NB channel. This suggests that the streptavidin used to detect NB also detects endogenous biotin which may be naturally filling the pockets.

**FIGURE 3:**
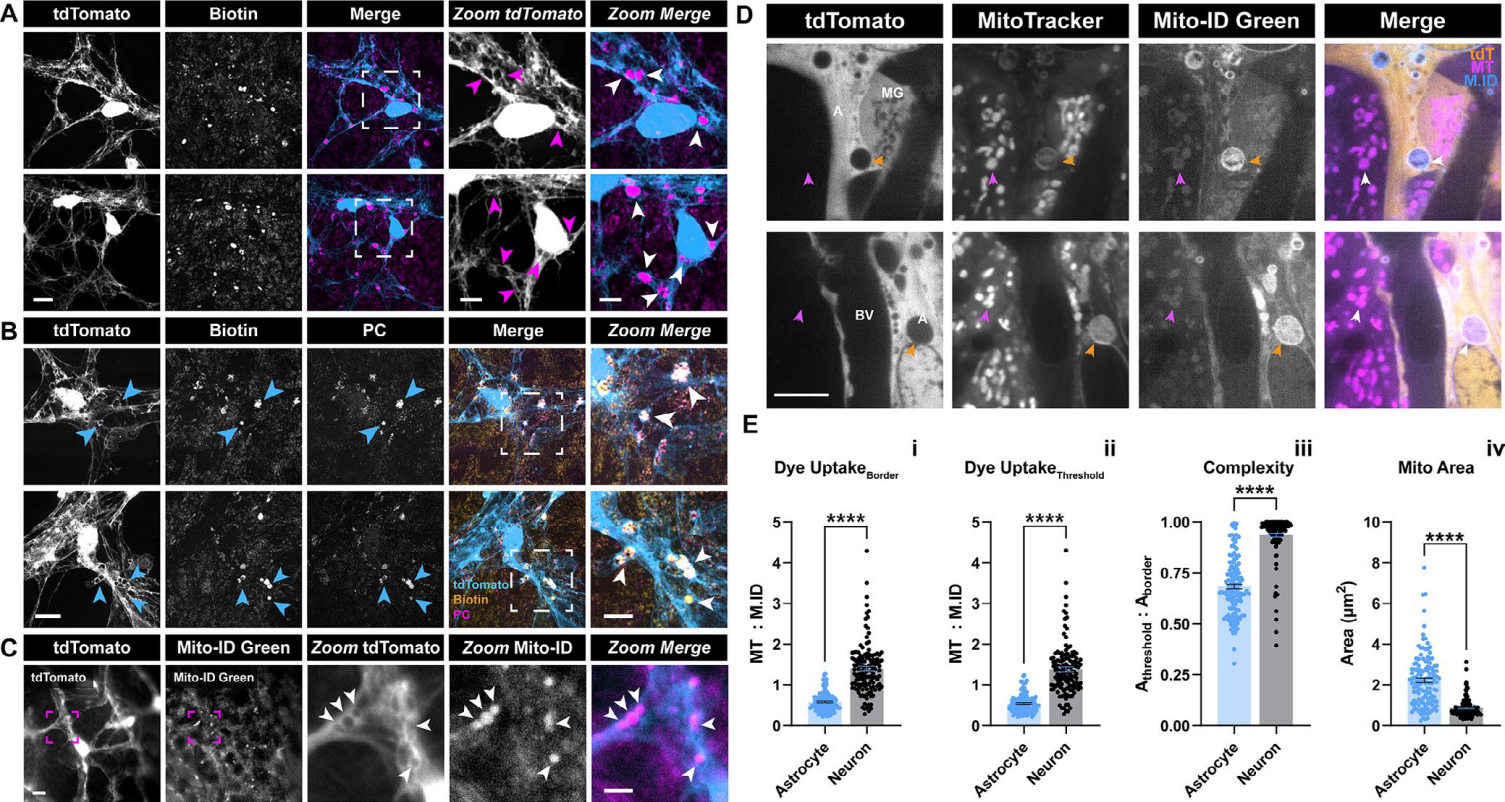
Astrocyte pocket mitochondria are anaplerotic and depolarized. Pockets label for endogenous biotin due to the high local density of mitochondria. (A) 280× confocal images of astrocytes (tdTomato) from retina labeled with streptavidin‐647 to reveal endogenous biotin. Biotin clusters into pockets that are devoid of tdTomato. Arrows indicate regions where pockets are clearly filled with biotin. (B) Biotin labeling in pockets is due to biotin‐containing mitochondrial enzymes. 280× confocal images of astrocytes (tdTomato) co‐labeled for endogenous biotin using fluorescent streptavidin and pyruvate carboxylase (PC). Both markers colocalize with one another and tdTomato void regions. Arrows indicate exemplar pockets. (C) Live imaging of astrocytes ex vivo at 40× magnification shows colocalization of the mitochondrial dye Mito‐ID Green in tdTomato‐negative regions of the cell. The labeling pattern closely resembles that of streptavidin‐biotin and PC labeling. Arrows indicate tdTomato‐negative regions filled with mitochondria. Scale is 10 μm for full‐size images and 5 μm for zoomed images A–C. (D) Ex vivo live retinal imaging at 280×. Astrocyte pocket mitochondria (orange arrow) take up less of the potential‐sensitive dye MitoTracker Deep Red FM than surrounding neuronal mitochondria (magenta arrow). Conversely, they uptake more of the potential‐insensitive Mito‐ID Green (marker of mitochondrial biomass) than neuronal mitochondria. Scale is 5 μm. (E) The ratio of MitoTracker (MT) to Mito‐ID (M.ID) is a measure of relative mitochondrial potential. This measure is higher in magnitude for neuronal mitochondria compared to astrocytic, irrespective of whether the entire mitochondrial border is used as ROI (I, 2.4×, *Z*‐score −11.7) or if a thresholded area is used (ii, 2.5×, *Z*‐score −12). Both measures were quantified, since dye uptake was uniform in neurons but complex in astrocytes (iii, *Z*‐score −11.6). Overall mitochondrial area was higher per ROI in astrocytes than in neurons (iv, *Z*‐score 10.2). Statistics are presented for Mann–Whitney *U* tests, as data were not normally distributed. All measures had *p* < 0.0001; *N* = 131 mitochondrial ROIs each from 10 fields of view for both groups.

When tdTomato‐expressing astrocytes are labeled using only fluorescently tagged streptavidin, the signal is extremely strong within the pockets, showing that they do have high levels of endogenous biotin (Figure 3A). In mammals, biotin is used as a coenzyme for five carboxylase enzymes: acetyl‐CoA carboxylase α (ACC1), acetyl‐CoA carboxylase β (ACC2), pyruvate carboxylase (PC), methylcrotonyl‐CoA carboxylase (MCC), and propionyl‐CoA carboxylase (PCC). Except for ACC1, all are located within mitochondria. To test whether astrocyte pockets contain at least one of these as a surrogate marker for mitochondria, we double‐labeled for biotin and PC (Figure 3B). The presence of PC identifies these mitochondria as anaplerotic, supporting intermediates of the tricarboxylic acid (TCA) cycle. As further confirmation, we incubated living ex vivo retina with Mito‐ID green and found the fluorescent signal localized to astrocyte pockets (Figure 3C). The cross‐sectional area of mitochondria identified in this way is quite large (2.15 ± 0.02 μm^2^, mean ± SEM, *n* = 87), much larger than expected in astrocytes and globular mitochondria observed in retinal neurons.

Pocket mitochondria are notable also in their resting potential (Ψ). We incubated and imaged ex vivo retina loaded with Mito‐ID Green and MitoTracker Deep Red FM (Figure 3D). Mito‐ID (M.ID) localizes to mitochondria in a potential‐independent manner, serving as a measure of total mitochondrial biomass. In contrast, MitoTracker (MT) enters mitochondria in a potential‐dependent manner. Dual labeling allows us to normalize the potential‐dependent signal to total biomass to compare voltage of mitochondria that differ in size. Compared to surrounding neuronal mitochondria, astrocyte mitochondria are relatively depolarized (Figure 3E). The ratio of MT:M.ID is ~2.5× higher in neuronal mitochondria compared to astrocytic. Assuming MT uptake reflects the Nernst potential, a 2.5‐fold difference in uptake translates to ~23 mV difference in polarization (ΔΨ). The combination of a relatively depolarized potential and high levels of PC suggest that these mitochondria are optimized for biosynthetic processes rather than oxidative phosphorylation.

**FIGURE 4:**
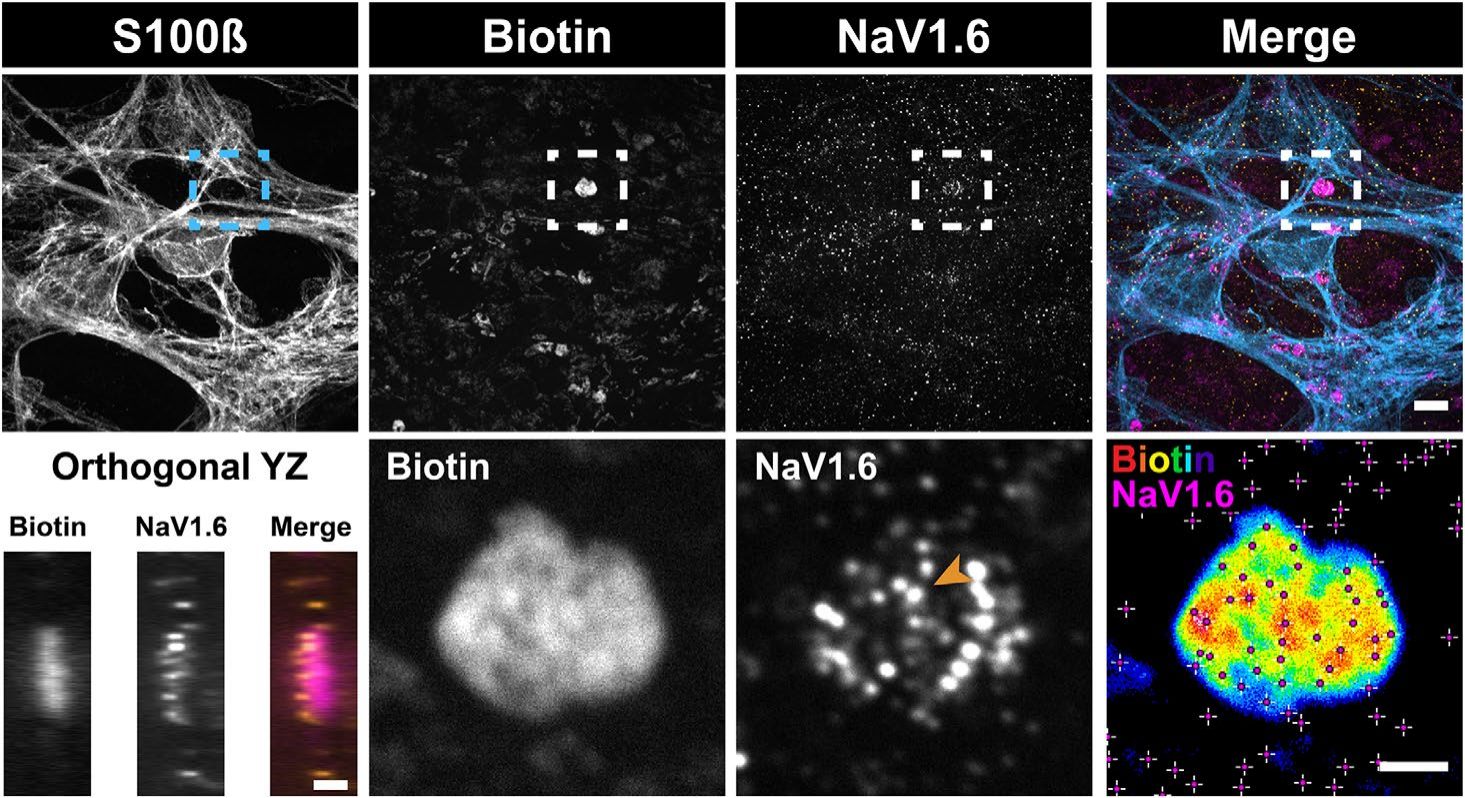
Na_V_1.6 puncta closely appose pocket mitochondria surfaces. 280× confocal images of astrocytes labeled for S100β, biotin, and Na_V_1.6. The Na_V_1.6 puncta preferentially localize to areas of high‐density biotin labeling within pockets. Zoomed regions (dashed boxes) show example mitochondrial/Na_V_1.6 pockets. Biotin intensity is revealed using a 16‐color heatmap (red is high intensity and blue is low). Na_V_1.6 locations are shown with magenta cross hairs. Orthogonal views correspond to the YZ plane taken at the orange arrow. Scale is 5 μm for full image (top row) and 1 μm for YZ and zoom images (bottom row). Using regions of high‐density biotin labeling within astrocytic volume as reference, we visually determined the probability that any Na_V_1.6 puncta colocalized to the tdTomato‐negative space housing mitochondrial biotin (pNa_V_|Biotin = 99.4%), the probability of high density Na_V_1.6 labeling within the same region (pNa_V__HD|Biotin = 73.5%), and the probability of biotin localizing to a tdTomato‐negative region (pTdT_null|Biotin = 100%). We quantified 972 pockets from 50 images and pooled across eccentricities. pNa_V__HD is less than pNa_V_ due to the presence of smaller pockets.

A recent paper described a Na_V_1.5 subpopulation closely associated with subsarcolemmal mitochondria in adult cardiomyocytes (Pérez‐Hernández et al. 2021). To investigate the structural relationship between sodium channel puncta and mitochondria, we labeled retinal astrocytes for S100β, biotin, and Na_V_1.6. Similar to cardiomyocytes, we find that Na_V_1.6 puncta are in close apposition to the mitochondrial surface (Figure 4), certainly within the depth resolution of our imaging system (~200 nm). We quantified the relationship between S100β/tdTomato void pockets, high density Na_V_1.6, and mitochondrial presence by scoring the probability of colocalization. We find that the probability of observing any Na_V_1.6 in a pocket labeled for biotin (pNa_V_|Biotin) is 99.4% and the probability of observing high density Na_V_1.6 labeling in these same regions (pNa_V__HD|Biotin) is 73.5%. The discrepancy is due to variation in the size of the pockets; small pockets are less likely to appear as containing high‐density puncta. Finally, as expected, the probability of biotin localizing to a tdTomato‐negative region (pTdT_null|Biotin) is 100%. These data show that the combination of anaplerotic mitochondria, high density Na_V_1.6, and volumes void of S100β define distinct metabolic compartments within retinal astrocytes. Notably, this labeling pattern was not observed with Na_V_1.1 or Na_V_1.8, which are known to be expressed in the retina as well (Figure S1).

**FIGURE 5:**
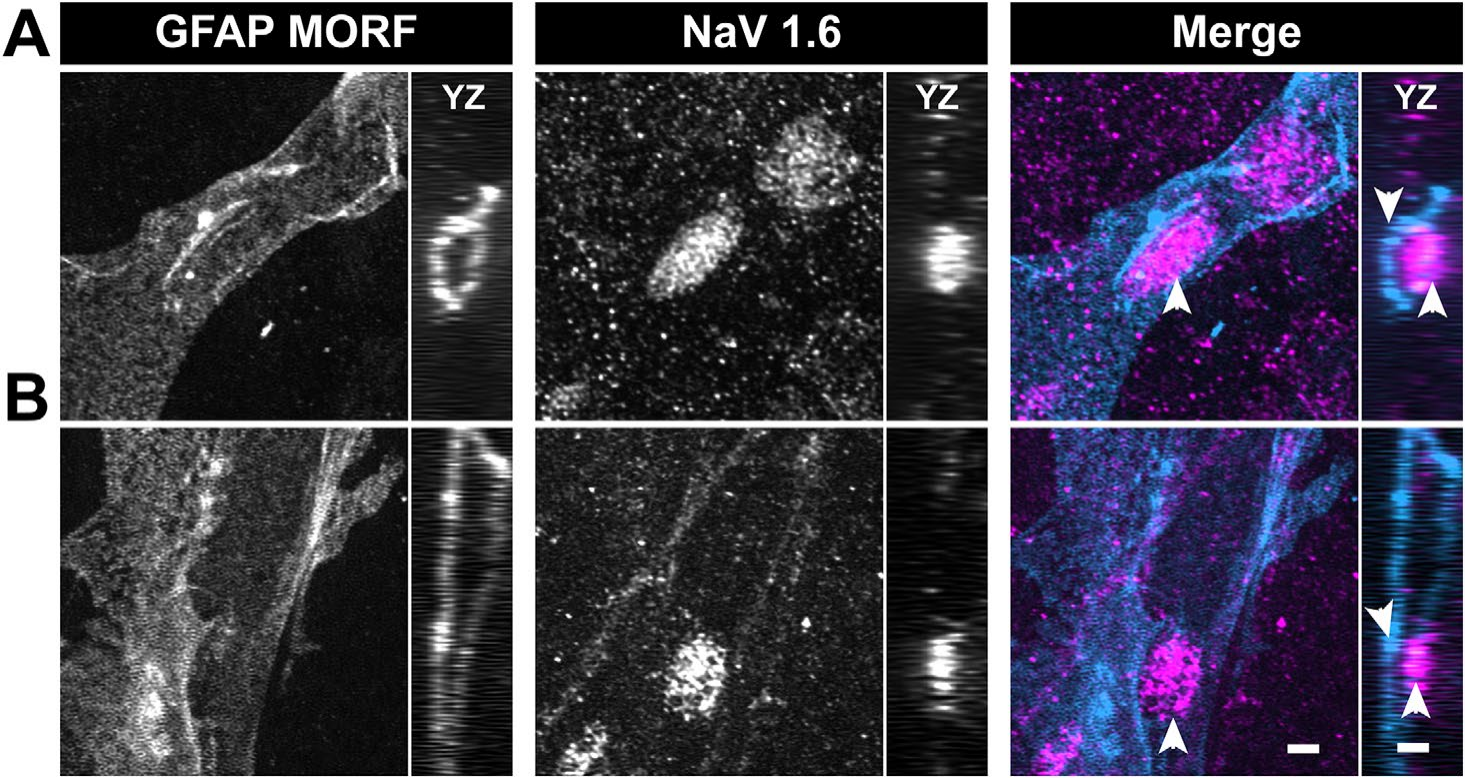
Pocket Na_V_1.6 is restricted to the membrane facing ganglion cell neurons. (A and B) Na_V_1.6 localizes asymmetrically within astrocyte pocket membranes. Pockets that form at immunohistochemically distinct inner‐ and outer‐retina facing membranes contain Na_V_1.6 only on the outer‐facing membrane and within the pocket. Example pockets with Na_V_1.6 (arrows) & corresponding YZ orthogonal views. Scale is 1 μm.

In examining Na_V_1.6 localization, we noticed that puncta only appear to form on the membrane facing the layer of RGC neurons and their axons, not toward the vitreous body of the eye (Figure 5). Retinal astrocytes are thin, forming functionally planar sheets less than a micron thick. Thus, for much of each astrocyte, this configuration was not easily discerned, given that the axial resolution of our imaging system is close to cell thickness (again ~200 nm). However, many processes and regions proximal to the cell body are sufficiently thick to form immunohistochemically distinct membranes where this observation could be made. This suggests sodium ions entering from RGCs may be important for sensing neuronal activity within astrocyte pockets.

**FIGURE 6:**
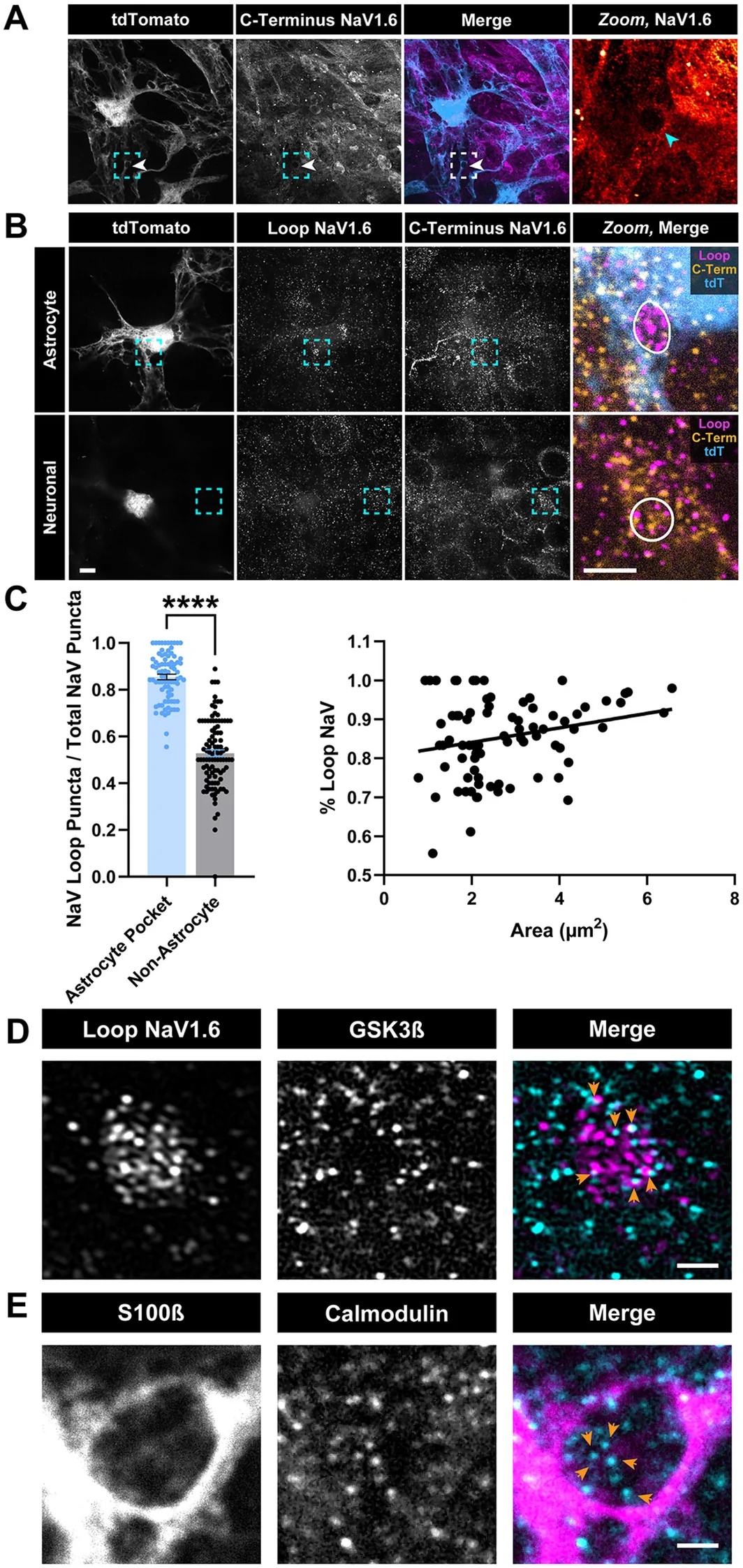
Pockets are enriched for Na_V_1.6 with epitope masked C‐terminal domains. (A) Unlike previous figures where Na_V_1.6 is labeled using an antibody against the cytosolic loop between domains II and III, here we label Na_V_1.6 using an antibody against the C‐terminal region, at residues 1854–1951. Labeling of Na_V_1.6 with this antibody is largely absent from nodes but diffusively labels outside of nodes. Arrows indicate regions of interest at nodes. Scale is 5 μm for full‐size images and 2.5 μm for zoomed regions. (B) 280×, single *z*‐slice confocal images of regions containing tdTomato‐expressing astrocytes and surrounding neuronal regions. Zoomed areas show either (i) pockets with enriched loop‐labeled Na_V_1.6 compared to C‐terminal labeled Na_V_1.6 or (ii) neuronal region with the same area and roughly equal labeling of both epitopes. Scale is 1 μm. (C) Quantification of 10 confocal images like those in panel B, showing significant exclusion of C‐terminal tail labeling in astrocyte pockets compared to non‐astrocytic regions (*p* = 0.0006). As pockets increase in size, the probability of observing C‐terminal‐labeled Na_V_1.6 decreases (95% confidence interval for slope [0.001485, 0.03578], *R* ^2^ = 0.06). (D) Shows GSK3β in pockets demarcated by Na_V_1.6 and (E) shows calmodulin immunolabeling in pockets demarcated by S100β. Pockets are demarcated using different methods due to host antibody compatibility. Deconvolution was performed in D to better show close apposition of Na_V_1.6 and GSK3β puncta. All images are single *z*‐slice. Scale is 1 μm.

All Na_V_1.6 labeling shown so far is the result of antibodies that target an epitope on the cytosolic loop between domains II and III. Remarkably, the labeling pattern using an antibody targeting the C‐terminal domain at residues 1854–1951 in addition was completely different (Figure 6A). Antibodies targeting the C‐terminal domain largely fail to reveal high density Na_V_1.6 within pockets. This is not an artifact of the C‐terminal domain‐targeting antibody, which successfully labels neurons in the RGC layer and also diffusely labels throughout the astrocyte volume (Figure 6A,B). When comparing astrocyte pockets with randomly selected, equivalent volume regions of RGCs, the ratio of C‐terminal domain labeling to loop domain labeling was significantly different (*p* = 0.0006; Figure 6C). The likely explanation is that astrocyte pockets are enriched for C‐terminal domain epitope masked Na_V_1.6. We found in additional labeling experiments that both GSK3β and calmodulin appear in astrocyte pockets (Figure 6D,E). Moreover, we verified that the lack of C‐terminal domain labeling was not due to a lack of expression of *Scn8a* transcripts encoding a C‐terminal domain. This was accomplished by parsing bulk RNA sequencing data from astrocyte enriched and non‐enriched retinal tissue from (Cullen et al. 2024). The one mouse transcript that lacks encoding for a C‐terminal domain (×10) is not expressed in retinal astrocytes or the retina in general (Figure S2). Additionally, the C‐terminal domain and loop domains are far enough away in 3D space that it is unlikely that steric effects of known binding interactions with the C‐terminal domain by GSK3β or calmodulin would affect the accessibility of the loop domain epitope (Figure S3).

**FIGURE 7:**
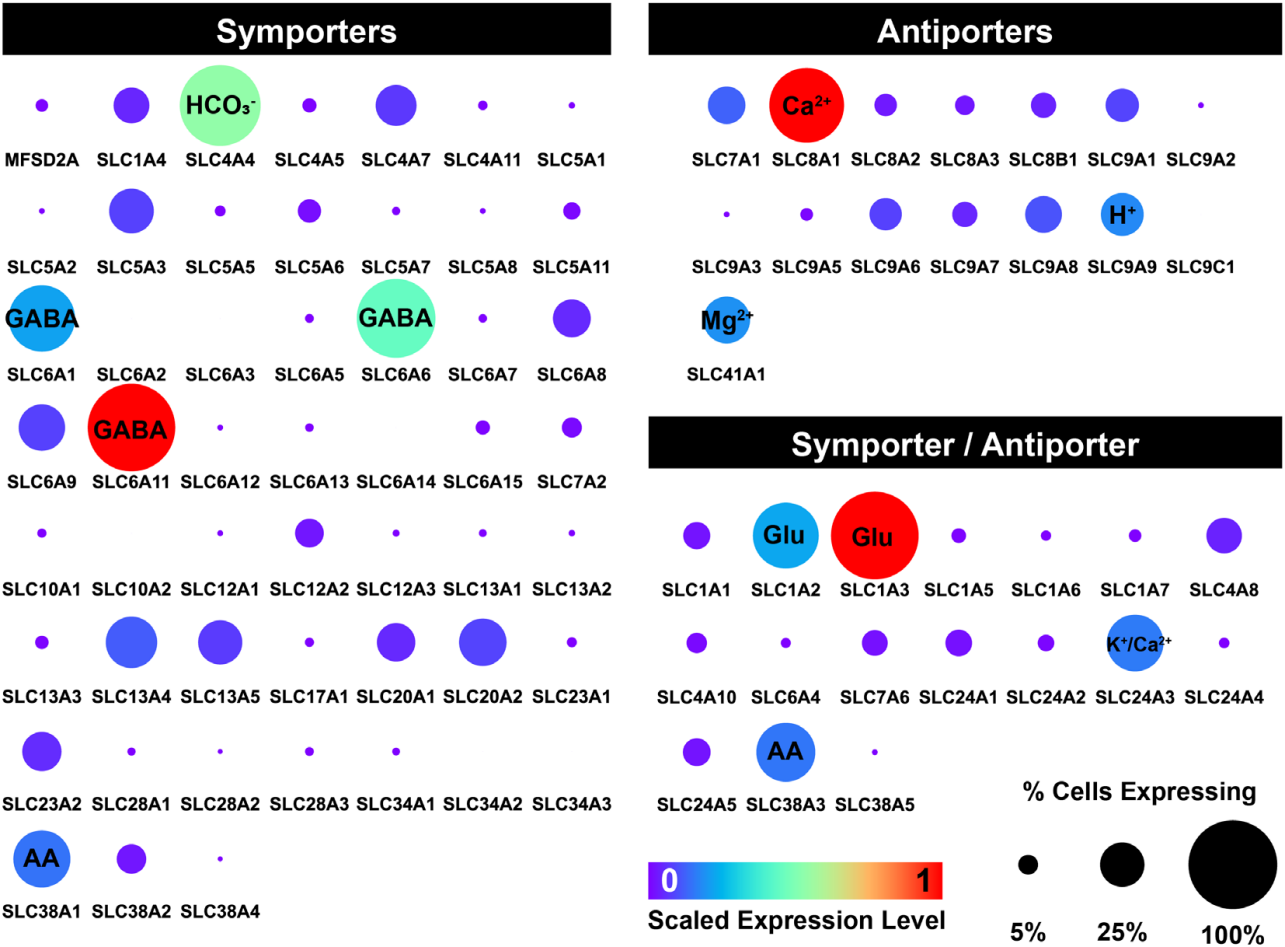
scRNA expression of sodium‐dependent transporters in retinal astrocytes. Dot plots show scRNA expression data from the open‐source Mouse Retinal Cell Atlas (MRCA, Li et al. 2024). Expression data is restricted to retinal astrocytes. Circle size corresponds to the percent of cells which express each gene, and the color indicates scaled expression within each group. Prominent sodium symport activity includes GABA, bicarbonate, and glutamate transport. Prominent antiport activity involves calcium, magnesium, and proton transport. AA stands for amino acid, and Glu is glutamate.

Apart from its role in affecting cell voltage, sodium is often coupled with the transport of ions and metabolites across the cell membrane. Sodium entry through Na_V_1.6 could trigger either the efflux of metabolites through a symporter or the influx of metabolites through an antiporter (or could inhibit normal operation of either). To identify likely candidates, we searched open‐source single‐cell RNA sequencing data for mouse retinal astrocytes for expression of all mouse genome‐encoded transporters that involve sodium (top candidates shown in Figure 7) (Li et al. 2024). Prominently expressed symporter genes involve transport of bicarbonate (*Slc4a4*), GABA (*Slc6a1, Slc6a6*, and *Slc6a11*), and glutamate (*Slc1a2* and *Slc1a3*), while the most prominent antiporter gene involves the transport of calcium (*Slc8a1*).

**FIGURE 8:**
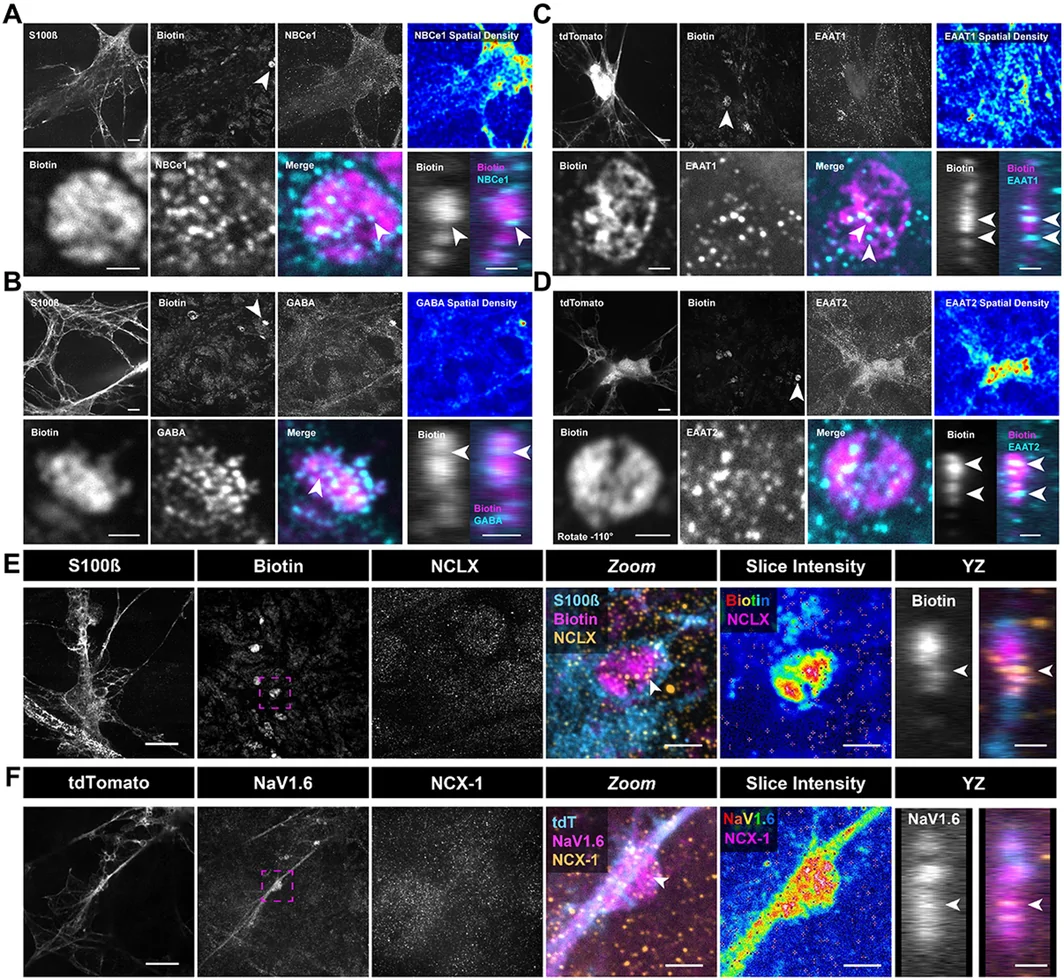
Sodium‐coupled symport in astrocytes: scRNA sequencing shows high expression of a variety of symporters which transport sodium along with other molecules. The highest and most ubiquitously expressed of these genes are implicated in the transport of bicarbonate, GABA, and glutamate. (A) At the protein level, we find high expression of NCBe1 (bicarbonate transporter, gene SLC4A4). Intensity‐weighted spatial density of NCBe1 puncta shows prominent enrichment in astrocytes. While present in pockets, there is not obvious enrichment there compared to the rest of the cell. (B) GABA transporters 1 and 3 (SLC6A1 and SLC6A11) as well as the taurine/GABA transporter SLC6A6 are highly expressed at the mRNA level. Using an antibody to localize GABA specifically, we find obvious enrichment in astrocytes. This enrichment is often very prominent in pockets. Within pockets, GABA colocalizes with both biotin signal and outside its bounds, indicating GABA is likely both within mitochondria and present in the pocket cytosol. (C and D) Both glutamate transporters EAAT1‐2 (SLC1A2 and SLC1A3) are present at the protein level in astrocytes but enrichment is not as prominent as observed for NCBe1 and GABA. Like NCBe1, EAATs are not found at noticeably higher densities in pockets compared to the rest of the cell. EAATs localize to both biotin‐positive and negative regions within the pocket. Spatial density was measured by a gaussian blur with sigma = 20 for a grid of intensity‐weighted maxima found in Fiji. Scale indicates 5 μm for full size images and 1 μm for zoom and YZ views (A–D). Both mitochondrial (NCLX, E) and plasma membrane (NCX, F) sodium calcium exchangers are present in retinal astrocytes in addition to the ganglion cell and nerve fiber layers in general. Both markers localize to pockets identified by biotin or high‐density Na_V_1.6. No obvious enrichment occurs in pockets compared to the rest of the cell. Scale is 10 μm in full‐size images, 2 μm in zoom, and 1 μm in orthogonal YZ views (E and F).

Because the majority of highly expressed genes involve symport (unidirectional membrane transport of two or more entities), we labeled retinal astrocytes for bicarbonate transporter NBCe1 (*Slc4a4*), GABA, and both excitatory amino acid transporters for glutamate, EAAT1/2 (Figure 8A–D). All transporters and GABA were abundant in astrocytes, which aligns with the scRNA sequencing data. While NCBe1 and EAAT1/2 were found throughout the cells (including pockets), they were not *enriched* in the pocket regions. However, GABA distributed not only throughout the astrocyte but also accumulated at very high density within the pocket regions. We similarly examined expression of sodium calcium exchangers NCLX (mitochondrial) and NCX (plasma membrane). Both are expressed throughout the cell but without enrichment in pockets (Figure 8E,F).

**FIGURE 9:**
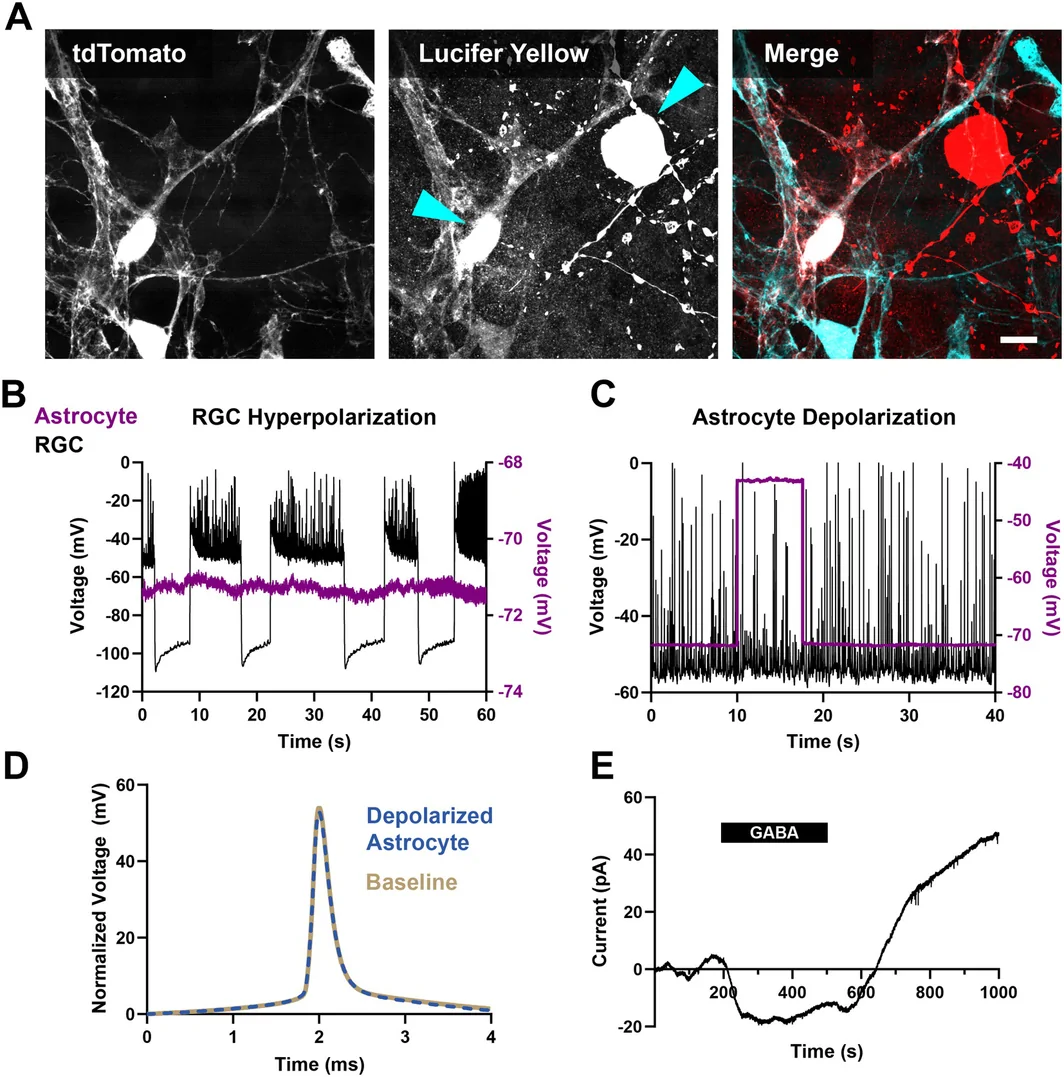
Astrocyte depolarization does not induce changes in RGC spike rate or action potential waveform measured at the cell body. (A) 280× confocal image of dual patch recording of an astrocyte (tdTomato, Lucifer Yellow) and OFF‐Sustained RGC (OFF‐S; Lucifer Yellow). Cyan arrows indicate cell bodies of patched cells. Scale is 10 μm. Data for representative traces were from this paired recording (B–D). (B) Current injection to hyperpolarize an OFF‐S RGC does not induce noticeable voltage change in paired astrocyte. Similarly, (C) induced depolarization of the astrocyte does not change the spike frequency of the RGC or (D) the waveform of action potentials observed at the RGC body. Spikes/second were the same during the period of astrocyte depolarization as at baseline. Curves in D are averages of an aggregated set of spikes during a period of astrocyte depolarization (great than the Na_V_1.6 activation threshold) and also during a baseline period where astrocyte potential is not manipulated. Potential was fixed to a starting voltage of 0 mV for all traces and interpolated to the same time duration. (E) Extracellular GABA induces an inward current in astrocytes which is reversed on GABA wash out. *N* = 3 unique pairs of astrocyte‐OFF RGC cells.

Because of the enrichment of GABA within pockets, we considered the possibility that sodium influx acts as a signal for the efflux of GABA. This might occur as neuronal activity increases extracellular potassium enough to depolarize astrocyte pockets locally to allow for Na_V_1.6 to open and sodium to flow into the cell, possibly indicating a feedback loop between neuronal activity and release of GABA from the astrocyte. GABA transporters like GAT‐3 (*Slc6a11*) transport one zwitterionic GABA molecule, two Na^+^ ions and one Cl^−^ ion in the same direction across the plasma membrane. Movement of GABA into the cell is thus accompanied by depolarization, and GABA movement out of the cell would cause hyperpolarization. While we found that astrocytes are responsive to extracellular GABA application, indicating functional GAT, in preliminary experiments warranting a larger sample size, we did not find a connection between either (1) modulation of RGC spike rate on astrocyte voltage or (2) modulation of astrocyte voltage on RGC spike rate or action potential waveform (Figure 9).

## 4 Discussion

Here we report a novel metabolic pocket‐like compartment within retinal astrocytes. These pockets are defined by (1) the absence of the cytosolic marker S100β (and by extension cytosolic fluorescent protein like tdTomato), (2) the presence of large, anaplerotic, and slightly depolarized mitochondria in close proximity to high density, C‐terminal domain masked, voltage‐gated sodium channel Na_V_1.6, and (3) high density localization of GABA. The most striking feature of these compartments is high density localization of Na_V_1.6 at levels not dissimilar to densities observed in neurons (Akin et al. 2016). To our knowledge, this study is the first to show distinct clustering of Na_V_1.6 in naïve astrocytes, compared to pan‐cellular expression in astrocytes of the brain in response to injury or disease (Liu et al. 2023). Previous studies have indicated high likelihood of voltage‐gated sodium channels on retinal astrocytes from electrophysiological recordings (Clark and Mobbs 1994; Newman 1985), and abundant evidence indicates their expression in astrocytes of other regions of the central nervous system (Sontheimer et al. 1996). In spinal cord astrocytes, for example, voltage‐gated sodium channels couple with sodium‐potassium ATPase, providing influx of sodium to keep the pump active (Sontheimer et al. 1994).

An important question is how Na_V_1.6 might function in retinal astrocytes given their hyperpolarized resting membrane potential (−74 mV), which is even more extreme when corrected for liquid membrane potentials (to approximately −87 mV), their small voltage‐response to light (1–2 mV), and the fact that Na_V_1.6 opens around −55 mV (Figure 9 (Holden, Boal, et al. 2025)). We think a potential explanation lies in the geometry of the pockets themselves. Astrocytes are incredibly thin as they blanket axons within the retinal nerve fiber layer, only a few hundred nanometers at most. Moreover, the pockets themselves appear to be isolated regions that are restrictive in the entry of proteins like S100β and tdTomato, which suggests size‐dependent barriers to free diffusion. The cytosolic volume is further reduced by the presence of exceptionally large mitochondria. These factors create a small, possibly electrically isolated volume within the cell that would respond more dramatically (and quickly) to changes in extracellular ion concentrations than might be expected of the overall cell. Because astrocytes are highly permeable to potassium ions, a relatively small change in extracellular potassium concentration could cause large changes in intracellular concentration, especially for small permeable volumes such as pockets. Given the potassium siphoning provided by Müller glia and how these cells influence astrocyte physiology and outer retina function (Holden, Boal, et al. 2025), such a possibility requires direct exploration, as does the influence on blood flow through astrocyte modulation (Holden et al. 2023). These more dramatic swings in voltage may depolarize the pocket enough to open the Na_V_1.6 channels. Alternatively, a depolarizing current might be needed to initially open the sodium channels. A very likely source could be neurotransmitter transporters like GAT‐3, which bring GABA into the cell along with two Na^+^ and one Cl^−^ for a net depolarizing effect. Uptake of GABA might trigger opening of Na_V_1.6, allowing a large inward current of sodium. This might also serve to stop further GABA uptake. This too is an important question, given GABA efflux from pockets could influence microglial activation state, especially in disease models. On this note, an important future direction is how diseases that influence RGC axon function might alter pocket expression of Na_V_1.6, GABA, and the anaplerotic state of mitochondria within the pockets.

Our results also indicate potential (though as of yet, indemonstrable) functional specialization of the Na_V_1.6 within pockets. We found that antibodies that include targeting an intracellular loop between domains II and III robustly reveal high density Na_V_1.6 labeling within the pockets whereas C‐terminal domain targeting antibodies do not (Figure 6). The most likely explanation is that the C‐terminal domain of Na_V_1.6 is bound by some protein–protein interaction. The IQ domain (residues 1893–1922) is within the region targeted by our C‐terminal domain antibody (immunogen corresponding to residues 1854–1951). In the literature, multiple protein–protein interactions that occur at the Na_V_1.6 IQ domain are reported, including with GSK3β and calmodulin. Interestingly, association of Na_V_1.6 and GSK3β is known to induce channel clustering (Baumgartner et al. 2025). Association of both GSK3β and calmodulin also have the reported effect of enhancing persistent sodium currents and affecting inactivation kinetics of the channel (Paul et al. 2016; Chichili et al. 2013; Scala et al. 2018). These data suggest that interactions at the Na_V_1.6 C‐terminal domain may be responsible for the clustering of Na_V_1.6 in the pockets and lead to persistent sodium currents to maintain the sodium potassium ATPase. We also show that known interactions of Na_V_1.6 with beta subunits, calmodulin, and GSK3β likely do not block access of the loop between domains II and III (Figure S3). An interesting aside is that in the Mouse Retinal Cell Atlas, we did not find astrocytes to express Na_V_1.6 mRNA robustly despite our definitive immunolabeling (Li et al. 2024). Thus, it is likely that Na_V_1.6 transcripts are docked within pockets and translated locally rather than from the perinuclear cell body. Preparations of single cell suspensions for use in atlases could lead to shearing of delicate astrocyte processes and thus to missing transcripts.

Close association of voltage‐gated sodium channels and mitochondria is a phenomenon observed in heart (Pérez‐Hernández et al. 2021). In cardiomyocytes, Na_V_1.5 is found in clusters in close apposition to subsarcolemmal mitochondria. Interestingly, in that study, the Na_V_1.5 antibody did not target the C‐terminal domain; *Scn5a* IQ domain is at residues 1843–1875 whereas the antibody Sigma #S0819 targets residues 493–511. Similar protein–protein interactions that drive clustering and activity of Na_V_1.5 in the heart might also occur in the retina. Pérez‐Hernández et al. suggest that influx of sodium from Na_V_1.5 leads to calcium extrusion through mitochondrial NCLX. While we show that astrocyte mitochondria express NCLX and the plasma membrane contains NCX, further characterization is needed to determine if similar phenomena occur in the retina. The fact that (1) calcium‐binding calmodulin is known to interact with Na_V_1.6 IQ domain, (2) calcium‐binding S100β is excluded from pockets, and (3) Na_V_1.5‐mitochondrial complexes are reportedly involved in calcium extrusion from mitochondria point to a possible calcium‐based signaling cascade in astrocyte pockets as well.

Our observation of high‐density punctate labeling of GABA within pockets points to its importance in pocket physiology. Our initial idea was that potassium increases from RGC spiking would depolarize the pocket, allowing Na_V_1.6 channels to open; influx of sodium would cause efflux of GABA through GAT‐3 symporter activity. This idea is consistent with our observation of high levels of pyruvate carboxylase in pocket mitochondria (Figure 3). Pyruvate carboxylase is an anaplerotic enzyme, meaning it is involved in regenerating an intermediate in a cycle that is being removed. Its role is to convert pyruvate into oxaloacetate. Oxaloacetate could be the TCA cycle intermediate that is removed from the mitochondria (by means of aspartate) and metabolized through the putrescine pathway to GABA. A key enzyme in this pathway (ODC1) is expressed in retinal astrocytes (Li et al. 2024). GABA could dock at GAT‐3, awaiting influx of sodium to trigger export. Additionally, depolarization of the pocket to open Na_V_1.6 would be similarly expected to move the voltage past the reversal potential of chloride (around −63 mV). scRNA data demonstrates retinal astrocytes express ClC chloride channels that bring two Cl^−^ into the cell with antiport activity of a single H^+^ (Li et al. 2024). Chloride entering the cell would aid in the export of GABA. The export of H^+^ ions would increase cellular pH which could be compensated by activity of NCBe1 bicarbonate transporter, which our immune labeling shows is prominent in the pockets and the astrocyte membrane in general. These ideas are illustrated below (Figure S4).

We did not find evidence that modulation of RGC spike rate caused any change in astrocyte voltage (Figure 9), as might be expected if extracellular potassium concentration were tied to the efflux of GABA. Conversely, injection of depolarizing current into astrocytes bringing their potential to a more positive voltage than required to open Na_V_1.6 did not affect RGC spike rate or action potential waveform as measured at the cell body, though astrocytes themselves were sensitive to extracellular GABA. Because of this, we believe the more likely explanation for GABA in pockets is simply due to cellular energetics and the normal role of astrocytes in neurotransmitter uptake. It is more likely that astrocytes take up GABA from the extracellular space and that GABA clusters in pockets because of the presence of mitochondria (Figure S4). In *Drosophila* neurons, hyperactive mitochondria sequester GABA through the activity of the mitochondrial transporter Aralar (Kanellopoulos et al. 2020). Given our observations of (1) high levels of pyruvate carboxylase and (2) relatively depolarized mitochondria within pockets, we conclude that mitochondria are metabolically active to support biosynthetic processes rather than oxidative metabolism. The combination of a high biosynthetic load and energetic demands involved with ion concentration balancing of active Na_V_1.6 make it likely that an Aralar‐dependent mechanism like that in *Drosophila* brains is also active in retinal astrocytes to sequester GABA. Astrocyte‐specific knockout of Na_V_1.6 could help inform this and other inferences from our data. GABA could then be broken down into succinate and enter the TCA cycle as a fuel source. It is possible that GABA efflux also occurs as a feedback mechanism on neural activity. The ability to patch an astrocyte, RGC cell body, and that RGC's axon would allow one to determine if GABA is released and active at ectopic, axonal GABA receptors, which could have consequences for neurotransmission.

## Author Contributions

D.J.C. provided resources, supervision, project management, and manuscript editing. J.M.H. conceptualized the study, conducted the experiments, analyzed the data, and wrote the manuscript.

## Funding

Experiments were performed with funds from National Eye Institute grant 5R01EY024997, and EY08126 (DJC) and in part through the use of the Vanderbilt Cell Imaging Shared Resource (supported by National Institutes of Health grants CA68485, DK20593, DK58404, and DK59637 to DJC).

## Conflicts of Interest

The authors declare no conflicts of interest.

## Supporting information


**Figure S1:** S100β‐devoid pockets do not contain Na_V_1.1 or Na_V_1.8. High‐magnification confocal images of astrocytes labeled for S100β, biotin, and Na_V_ subunits. S100β‐devoid pockets contain endogenous biotin indicative of mitochondria but not detectable Na_V_1.1 (top row) or Na_V_1.8 (middle row) compared to positive signal for Na_V_1.6 (bottom row). Scale is 1 μm in each row.

**Figure S2:** Both mouse retina and astrocytes specifically do not express the mRNA variant of SCN8a (Na_V_1.6) which lacks a C‐terminal domain. Lack of C‐terminal binding of Na_V_1.6 antibody in astrocytic nodes is not due to mRNA transcript variation. (i and ii) Bulk RNA sequencing data adapted from Cullen et al. (2024) show Na_V_1.6 transcript heterogeneity in mouse astrocytes (GFAP Cre−Ribotag + micro‐dissection) vs. whole retina. Predominant transcripts include Variant 1 (NM_001077499.2) and 2 (NM_011323.3). (iii) The sequence for the Human C‐terminal Na_V_1.6 immunogen (IQ domain) used in the Novus antibody is compared with all mouse Na_V_1.6 variant protein sequences available on NCBI. All sequences are identical except for (1) a K to R substitution in the Variant 1 reference sequence and (2) the fact that variant X10 lacks the immunogen sequence in its C‐terminus. Variant X10 is not expressed in the retina. TPM stands for transcripts per million and are recorded from Salmon output files.

**Figure S3:** Known protein–protein interactions with Na_v_1.6 likely do not sterically limit access to loop epitope. (A) Superimposed Cryo‐EM structure (pdb_00008gz1) of Na_v_1.6 (yellow) and associated β1 (red) and β2 (magenta) subunits with AlphaFold model of full‐length Na_v_1.6 (AF‐Q9WTU3‐F1, blue). Green highlights the C‐terminal domain of Na_v_1.6 while orange shows the loop region between domains II and III. (B) AlphaFold model of Na_v_1.6 with the same regions colored in A. Superimposed is the crystal structure of calmodulin (pdb_00003wfn, magenta), aligned using the IQ domain in the C‐terminal tail of Na_v_1.6. (C) AlphaFold model of Na_v_1.6 with the same regions colored in A/B. Magenta shows an AlphaFold structure of GSK3β with predicted interaction site at the C‐terminal domain of Na_v_1.6 using Haddock 2.4. Residues 1898 and 288 in Na_v_1.6 and GSK3β, respectively, were set as active, following predictions of Baumgartner et al. (2025).

**Figure S4:** Two hypotheses for pocket function. Astrocyte pocket with known components labeled in green (IHC or scRNA data). Model (A) (1) Krebs' cycle intermediate oxaloacetate is metabolized into aspartate and extruded from mitochondria, (2) Aspartate is metabolized eventually into GABA, which docks to GABA transporters, (3) Loss of oxaloacetate is compensated by its synthesis from pyruvate using pyruvate carboxylase, (4) Neural activity increases extracellular potassium, (5) Shift in potassium concentration depolarizes the astrocyte pocket, (6) Depolarization in the pocket allows Na_V_1.6 to open and sodium flows in, further depolarizing, (7) Depolarization also shifts past chloride reversal potential of −63 mV and chloride flows into the cell and H+ leaves, (8) Loss of H+ increases the pH of the pocket, so NCBe1 drives out bicarbonate and Na+. Loss of bicarbonate drives its equilibrium to produce H+, (9) GABA transporter runs in reverse, aided by high concentration of GABA and influx of Na+ and Cl^−^, (10) GABA reduces neural activity as feedback. Model (B) (1) GABA is taken up by the astrocyte through GAT and TauT, depolarizing the cell, (2) Depolarization in the pocket allows Nav1.6 to open and sodium flows in, further depolarizing the cell (acts as sodium boost), (3) Influx of sodium allows NaK ATPase to run smoothly to maintain potassium gradient, (4) GABA enters mitochondria and is metabolized for energy through the GABA Shunt, (5) ATP from GABA influx allows for NaK ATPase to keep running, (6) ClC and NCBe1 maintain pH and chloride gradients due to GAT activity. The mitochondria are not solely for this process, they are anaplerotic/biosynthetic for the whole cell. They are docked in the pocket for convenience.

## Data Availability

The data that support the findings of this study are available from the corresponding author upon reasonable request.
